# Arginase-1 in Plasma-Derived Exosomes as Marker of Metastasis in Patients with Head and Neck Squamous Cell Carcinoma

**DOI:** 10.3390/cancers15225449

**Published:** 2023-11-16

**Authors:** Linda Hofmann, Malgorzata Harasymczuk, Diana Huber, Miroslaw J. Szczepanski, Grzegorz Dworacki, Theresa L. Whiteside, Marie-Nicole Theodoraki

**Affiliations:** 1 Department of Otorhinolaryngology, Head and Neck Surgery, University of Ulm, 89075 Ulm, Germany; 2 Hillman Cancer Center, University of Pittsburgh Medical Center (UPMC), Pittsburgh, PA 15232, USA; 3 Department of Clinical Immunology, University of Medical Sciences, 61-701 Poznan, Poland; 4 Department of Biochemistry, Faculty of Medicine, Medical University of Warsaw, 02-097 Warsaw, Poland; 5 Department of Pathology, University of Pittsburgh School of Medicine, Pittsburgh, PA 15213, USA; 6 Departments of Immunology and Otolaryngology, University of Pittsburgh School of Medicine, Pittsburgh, PA 15213, USA; 7 Department of Otorhinolaryngology, Head and Neck Surgery, Klinikum rechts der Isar, Technical University Munich, 81675 Munich, Germany

**Keywords:** arginase-1, HNSCC, exosomes, metastasis

## Abstract

**Simple Summary:**

Head and neck cancer creates a highly immunosuppressive microenvironment, to which the enzyme Arginase-1 contributes. The impact of Arginase-1 on the clinicopathology and prognosis of head and neck cancer, however, is not fully understood. We evaluated Arginase-1 protein levels in both the tumors and plasma of patients. Patients with high tumor Arginase-1 levels had better clinicopathology and prognosis, while patients with high plasma Arginase-1 levels had unfavorable clinicopathology. We attributed this difference to the presence of Arginase-1-positive exosomes in the blood of patients. Exosomes, small vesicles for intercellular communication, are released by cancer cells and can travel through the body. Patients with high Arginase-1 levels in these exosomes had lymph node metastasis and poor prognosis. We conclude that exosomes can export Arginase-1 from the tumor to the periphery for further immunosuppression. Exosomal Arginase-1 might be a better indicator of metastatic disease and prognosis than tissue or plasma Argianse-1.

**Abstract:**

Immunoregulatory Arginase-1 (Arg-1) is present in the tumor microenvironment of solid tumors. Its association to clinicopathology and its prognostic impact are inconsistent among different tumor types and biological fluids. This study evaluated Arg-1 protein levels in tumors and the circulation of patients with head and neck squamous cell carcinoma (HNSCC) in relation to clinical stage and prognosis. Tumor Arg-1 expression was monitored via immunohistochemistry while plasma Arg-1 levels via ELISA in 37 HNSCC patients. Arg-1 presence in plasma-derived exosomes was assessed using Western blots in 20 HNSCC patients. High tumor Arg-1 expression correlated with favorable clinicopathology and longer recurrence-free survival (RFS), while high plasma Arg-1 levels were associated with unfavorable clinicopathology. All patients with low tumor and high plasma Arg-1 had nodal metastases and developed recurrence. This discrepancy was attributed to the presence of Arg-1-carrying exosomes. Arg-1 was found in plasma-derived exosomes from all HNSCC patients. High exosomal Arg-1 levels were associated with positive lymph nodes and short RFS. Circulating Arg-1^+^ exosomes represent a mechanism of active Arg-1 export from the tumor to the periphery. Exosomes reflected biologically relevant Arg-1 levels in metastatic HNSCC and emerged as potentially more accurate biomarkers of metastatic disease and RFS than tissue or plasma Arg-1 levels.

## 1. Introduction

Head and neck squamous cell carcinoma (HNSCC) accounts for more than 600,000 new cases annually worldwide and is the sixth most common cancer [1]. Despite significant advances in surgical procedures and the use of chemoradiation regimens, the survival remains poor, mostly due to metastatic disease and locoregional recurrence [2,3,4]. Most HNSCC patients have depressed anti-tumor immunity and a highly immunosuppressive tumor microenvironment (TME), which favor tumor escape from the immune system, contributing to tumor progression [5].

Several mechanisms facilitating tumor escape from immune surveillance have been described [6,7], among which Arginase-1 (Arg-1) plays an important role [8]. Arg-1 is a cytosolic enzyme originally isolated from the liver and present in the TME of most solid tumors [9,10], including HNSCC [11]. As part of the urea cycle, it is involved in the detoxification of ammonia and catalyzes the breakdown of the amino acid L-arginine to L-ornithine and urea [8,12]. L-ornithine is further metabolized to synthesize polyamines, which promote cell proliferation, migration and invasion. In addition to its direct effect on tumor growth, Arg-1 further fosters tumorigenesis by inducing immune suppression; it depletes arginine, which is essential for T-cell proliferation, in the TME [10]. Specifically, the presence of Arg-1 and L-arginine depletion was shown to down-regulate the expression of the T-cell receptor CD3ζ chain, thereby impairing T-cell functions [13,14,15]. Arg-1 is also highly expressed and secreted by macrophages and myeloid-derived suppressor cells (MDSCs) accumulating in the TME, leading to further arginine depletion and suppression of T- and NK-cell functions [16,17].

More recently, another immune-suppressive mechanism operating in the TME was identified. Tumor-derived exosomes (TEX), small-sized extracellular vesicles, mediate intercellular communication between the tumor and all other cells in the TME [18]. Exosomes accumulate in HNSCC patients’ plasma [19] and represent a mixture of TEX and non-TEX [20,21]. Increased plasma levels of exosomes correlate with disease activity and progression in patients with HNSCC [19,22]. We and others have shown that plasma-derived exosomes in cancer patients carry immuno-modulatory proteins and induce immune suppression in recipient immune cells [20,22,23].

This study evaluated the expression levels of Arg-1 in HNSCC tumors in situ and in the circulation of patients with HNSCC, in relation to clinicopathological data and the prognostic impact.

## 2. Materials and Methods

### 2.1. Patients and Samples

Two different cohorts of HNSCC patients were included in this study. The first cohort (for tumor and plasma analyses) consisted of primary tumor, adjacent normal mucosa and plasma from 26 patients (stage III/IV disease) undergoing surgery at the Otolaryngology University Clinic in Poznan, Poland (approved by the Ethics Committee at the University of Medical Sciences in Poznan), as well as primary tumor from 5 patients and plasma from 11 patients (stage I/II disease), obtained by Dr. M. Harasymczuk with permission from the Ethics Committee at the Poznan University of Medical Sciences, Poznan, Poland. Plasma from 10 control samples was collected from age- and gender-matched healthy donors (HDs; 9 male, 1 female, median age 57 years, range 40–71 years) at the Otolaryngology and Head and Neck Cancer Surgery Clinic at the University of Pittsburgh as per the IRB approval #960218. The second cohort (for exosome analyses) consisted of plasma from 20 HNSCC patients and 8 age- and gender-matched HDs (5 male, 3 female, median age 61 years, range 55–77 years) seen at the Department of Otorhinolaryngology, Head and Neck Surgery, Ulm University, Germany (approved by the Ethics Committee of Ulm University #90/15). All study subjects provided informed consent. The clinicopathological data of both patient cohorts are listed in Table 1.

Peripheral blood samples of HNSCC patients were collected before surgical treatment onset. Plasma for the first cohort was collected from peripheral blood via centrifugation at 300× *g* for 10 min; plasma for the second cohort was collected via centrifugation at 1000× *g* for 10 min, followed by 2500× *g* for 10 min. Plasma aliquots were stored at −20 °C.

### 2.2. Cell Lines

The HNSCC cell line PCI-13 (RRID:CVCL_C182) was established at the University of Pittsburgh Cancer Institute and was maintained as previously described [24]. The PCI-13 cell line originates from a poorly differentiated laryngeal carcinoma and was authenticated before use for the described experiments. Cells were cultured in DMEM medium supplemented with 10% (*v*/*v*) fetal calf serum (FCS), L-glutamine and antibiotics (all from Invitrogen, Carlsbad, CA, USA). Cells in log phase of growth were used for experiments and tested PCR-negative for mycoplasma and endotoxin (Mycoplasma Tissue Culture Detection Kit, Gen-Probe, San Diego, CA, USA and Limulus Amebocyte Lysate assay, Cambrex, East Rutherford, NJ, USA).

### 2.3. Antibodies

Anti-Arginase-1 Ab (sc-20150, RRID: AB_2058955, Santa Cruz Biotechnology, Santa Cruz, CA, USA) or isotype rabbit IgG control was used for immunostaining. In addition, through use of mouse anti-human pan-cytokeratin (CK) Ab (GA053, RRID: AB_2892089, Dako Omnis, Agilent, Santa Clara, CA, USA), the epithelial origin of tumor cells in tissue sections was verified. Working concentrations of Abs were 2–10 μg/mL.

### 2.4. Immunofluorescence

Cultured tumor cells were placed on glass slides and allowed to air dry. The cells were then washed in PBS, fixed in 2% (*w*/*v*) paraformaldehyde for 15 min, permeabilized with 0.1% Triton X, blocked with 2% bovine serum albumin (BSA) in PBS for 45 min and incubated with primary Abs for 1 h at room temperature (RT). The Cy3 donkey anti-goat secondary Ab (705-165-147, RRID: AB_2307351, Jackson Immuno Research, West Grove, PA, USA) was used at 1:1000 dilution, and cells were incubated for 45 min at RT in the dark. In controls, the primary Ab was replaced by 0.5% BSA. Sections were mounted in a medium with DAPI (Vector Laboratories, Burlingame, CA, USA) in order to visualize cell nuclei and were examined in a Nikon Eclipse E-800 (Nikon Instruments, Melville, NY, USA) fluorescence microscope.

### 2.5. Immunohistochemistry

Paraffin-embedded 4 µm thick tumor sections (4 μm thick) were prepared. Following the standard deparaffinization process and blocking of endogenous peroxidase activity, staining was performed using the Dako EnVision+ System, following the manufacturer’s guidelines. Briefly, tumor sections were incubated with rabbit anti-human Arg-1 overnight, followed by incubation with horseradish peroxidase (HRP)-conjugated anti-rabbit Ab and peroxidase substrate DAB (3,3′-diaminobenzidine). Tumor sections were then counterstained with Meyers hematoxylin and mounted in glycerol jelly.

### 2.6. Evaluation of Tissue Staining

Stained tissue sections were examined using an Olympus 700 light microscope (Olympus, Center Valley, PA, USA) at ×400 magnification. Adobe Photoshop version 7.0 software was employed for digital image analysis. Two investigators (GD and MH) independently assessed Arg-1 expression in tumor samples, examining five separate fields per section. The results were averaged, and scoring was based on both the percentage of positive tumor or immune cells per section and the staining intensity. Staining was categorized as follows: none (0), rare (≤25% cells stained), variable (26–75% cells stained) and positive (>75% cells stained). Staining intensity levels were determined empirically and designated as weak (1), moderate (2) or strong (3). Further, the localization of Arg-1^+^ cells in either tumor parenchyma or stroma was determined. The H scores, calculated as % positive cells x staining intensity, were used to assess Arg-1 expression levels in both tumor parenchyma and stroma. These H scores were utilized in statistical analyses to correlate expression with the clinicopathologic data and recurrence-free survival (RFS).

### 2.7. Effects of Arginase-1 on Tumor Cell Growth In Vitro

PCI-13 tumor cells (1 × 10^6^) were cultured in DMEM medium supplemented with 10% FBS, 100 IU/mL penicillin, 100 μg/mL streptomycin and L-glutamine (2 mmol/L) at 37 °C in an atmosphere of 5% CO_2_ in air. To certain wells, 1 mM/mL of L-arginine dissolved in ddH2O (Sigma Aldrich, St. Louis, MO, USA) and/or 200 µM/mL of N-hydroxy-L-arginine (nor-NOHA; Cayman Chemical, Ann Arbor, MI, USA), an arginase inhibitor, were added. Physiological human plasma concentrations of L-arginine were reported to be around 200 µmol/L [25], and we used supraphysiological concentrations for in vitro testing. After seven days of culture, the tumor cells were harvested and counted under microscope using 0.4% trypan blue to assess the proliferation rate and cell viability.

### 2.8. ELISA

Plasma was analyzed for levels of soluble Arg-1 via ELISA (RD182028100, BioVendor, Ashville, NC, USA). The assays were performed as recommended by the manufacturer with 50 µL plasma per well. The assay sensitivities were 0.5 ng/mL. All samples were tested in duplicate, and background-subtracted average values were recorded.

### 2.9. Plasma–Exosome Isolation via Size Exclusion Chromatography (SEC)

Exosomes from plasma were isolated as previously described [26]. Briefly, 1 mL pre-cleared (2000× *g* for 10 min at RT and 14,000× *g* for 30 min at 4 °C) and filtered (0.22 μm filter, Merck Millipore, Burlington, MA, USA) plasma samples was applied to a Sepharose 2B column and eluted with PBS. Fraction #4, containing majority of eluted exosomes [26], was collected. Exosomal protein concentration was quantified using Pierce BCA protein assay kit (Thermofisher Scientific, Waltham, MA, USA) and calculated as μg of protein per mL of input plasma volume.

### 2.10. Exosome Characterization

Exosome fractions #4 were characterized via transmission electron microscopy, nanoparticle tracking analysis and Western blot according to the minimal information for studies of extracellular vesicles (MISEV) recommendations [27] and as described previously [23] (EV-Track ID EV200068).

### 2.11. Western Blots

Exosomes were tested for the presence of Arg-1 and the exosome marker TSG101. Western blots were performed as previously described [23]. Briefly, exosomes were lysed in Lane Marker Reducing Sample Buffer (Thermofisher Scientific), separated on 12% Precast Protein Gels (Bio-Rad, Hercules, CA, USA), applying 20 μg protein/lane and transferred onto PVDF membranes using Trans-Blot Turbo (Bio-Rad). For detection of Grp94 and ApoA1, 20 µg of PCI-13 cell lysate and plasma (1:20 diluted in PBS) was used as positive controls. Membranes were blocked with 5% milk in TBS-T for 1 h at RT and incubated overnight at 4 °C with anti-Arg-1 Ab (1:1000, GTX109242, RRID: AB_2036264, GeneTex, Irvine, CA, USA) or with anti-TSG101 Ab (1:500, PA5-31260, RRID: AB_2548734, Thermofisher Scientific). Goat anti-rabbit HRP-conjugated secondary antibody (1:10,000, 31460, RRID: AB_228341, Thermofisher Scientific) was added for 1 h at RT, and blots were developed with ECL substrate (34076, Thermofisher Scientific) using the ChemiDoc XRS+ Imaging System (Bio-Rad). Within each antibody, exposure times were the same between separate membranes. Semi-quantitative analysis of Arg-1 band intensity was performed using Image Lab Software v 6.0.1 (Bio-Rad, RRID: SCR_014210).

### 2.12. Data Analysis

Correlations were calculated using non-parametric Spearman correlation. Differences between groups were calculated using the non-parametric Mann–Whitney test. *p* values < 0.05 were considered significant. The impact of tumor Arg-1 mRNA expression on overall survival in the TCGA cohort was analyzed using UCSC Xena Platform [28]. Assuming a non-linear relationship between tumor Arg-1 H score and recurrence-free survival, recursive partitioning analysis (decision tree model) was used to determine the tumor Arg-1 H score cut off for high recurrence risk. In order to not lower the modest sample size, robust and less outlier-sensitive median was used to group paired tumor–plasma samples and exosome samples into high vs. low Arg-1 expression. Kaplan–Meier plots with log-rank test were used to estimate RFS.

## 3. Results

### 3.1. Patients

Clinicopathologic data for all patients enrolled in the study are presented in Table 1. The initially recruited cohort (tumor, plasma) consisted of 37 patients (29 males and 8 females aged 47–78 years, median 60 years) with histopathologically confirmed primary squamous cell carcinomas. Histological tumor differentiation was moderate (G2) in 25 cases and poor (G3) in 9 cases. Tumor stages ranged from T1/2 in 11 cases to T3/4 in 26 patients, and 23 patients had nodal involvement. According to the 8th edition of the UICC, 11 patients had low-stage disease (I/II), whereas 26 patients had advanced disease (III/IV). Follow-up was performed for 4 years after therapy (surgery with adjuvant chemoradiotherapy). Initially, physical examination and scans were performed at 3-month intervals. After 2 years, scans were performed every 6 months. Tumor recurrence was documented for 13 patients during this time period, while 24 patients remained tumor free.

The separately recruited cohort of 20 patients used for exosome studies included 12 males and 8 females, ranging in age from 51 to 82 years (median 65 years). Further, 8 tumors were G2 and 7 were G3. Ten patients each had T1/2 and T3/4 stage disease, respectively, while ten patients had nodal involvement, twelve patients had early-stage (UICC I/II) and eight had advanced (UICC III/IV) disease. Tumor recurrence was documented for 6 patients, while 14 patients remained tumor-free in the follow-up period (mean 28 months).

### 3.2. Arg-1 Expression and Activity in PCI-13 Cells

**Figure 1:**
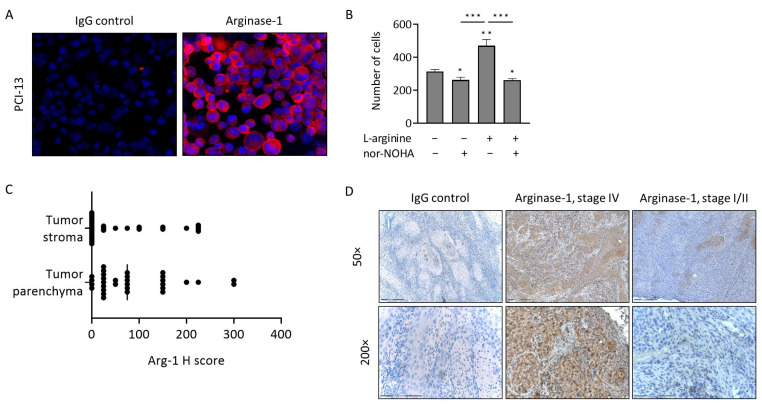
Arginase-1 (Arg-1) expression in head and neck squamous cell carcinoma (HNSCC). (**A**) Arg-1 expression in cultured PCI-13 cells, as determined by immunofluorescence (×400 magnification, pink = Arg-1, blue = nuclei). (**B**) In vitro tumor proliferation of PCI-13 cells in the presence of L-arginine and/or nor-NOHA, an Arg-1 inhibitor. Bars represent mean ± standard deviation (SD) of *n* = 3 replicates. *p*-values were determined by Mann–Whitney test, with *, ** and *** corresponding to *p* ≤ 0.05, *p* ≤ 0.01 and *p* ≤ 0.001, respectively. * above bars indicate significance to the untreated control (left bar). (**C**) Arg-1 expression in tumor stroma and parenchyma of *n* = 31 HNSCC patients, as determined by immunohistochemistry (IHC) and presented as H-score. (**D**) Representative IHC staining for Arginase-1 and IgG control of HNSCC stage IV and stage I/II tumor sections. Scale bar in 50× = 300 µm, scale bar in 200× = 150 µm.

Immunofluorescence showed that Arg-1 was expressed in the PCI-13 cells (Figure 1A). We examined how tumor cells utilize Arg-1 for the catalysis of exogenous arginine. PCI-13 cells were cultured in the presence of nor-NOHA (arginase inhibitor), L-arginine or the combination of both, and cell proliferation was assessed. Nor-NOHA alone, blocking the endogenous L-arginine turnover, slightly reduced cell proliferation (*p* = 0.014, Figure 1B). As expected, the addition of L-arginine to the culture strongly increased tumor cell proliferation relative to the untreated control (*p* = 0.002). The combined addition of L-arginine + nor-NOHA significantly inhibited arginine-dependent tumor growth (*p* = 0.0007), suggesting that Arg-1 enzymatic activity (i.e., arginine catalysis) and high Arg-1 expression levels regulate tumor cell proliferation. Thus, high Arg-1 levels in the tumor are expected to reflect highly active metabolism, rapid arginine utilization and rapid tumor growth.

### 3.3. Arg-1 Expression in Tumor Tissues

Immunostaining of 31 tumor specimens (cohort 1) indicated that Arg-1 was expressed in tumor tissues (parenchyma and stroma) at variable levels, as indicated by the H scores (Figure 1C, Supplementary Table S1). IHC staining for Arg-1 ranged from weak to strong in stage III/IV tumors, while it was negative or only weakly positive in stage I/II tumors (Figure 1D). There was a weakly significant correlation (r = 0.41, *p* = 0.025) between Arg-1 H scores in the tumor parenchyma and stroma (Supplementary Figure S1).

### 3.4. Arg-1 Expression in Tumor Correlates with Clinical Data and Recurrence-Free Survival (RFS)

**Figure 2:**
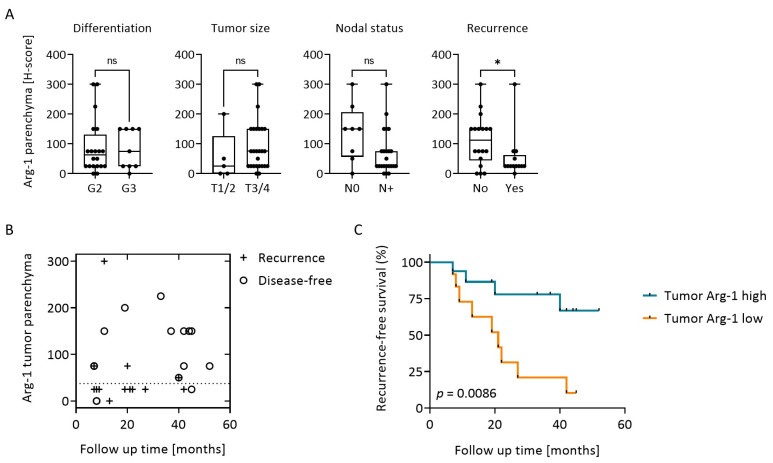
Tumor Arg-1 expression in relation to clinical data and recurrence free survival (RFS). (**A**) Arg-1 H scores in tumor parenchyma of *n* = 31 HNSCC patients, grouped according to tumor differentiation grade, tumor size, nodal status and development of recurrence. *p*-values were determined by Mann–Whitney test, with * corresponding to *p* ≤ 0.05 and ns = not significant. (**B**) Arg-1 H scores in the tumor parenchyma are plotted against the time of recurrence. Recursive partitioning analysis identified the score of 37.5 as the natural cut-off point for a high recurrence risk (dotted line). (**C**) Kaplan-Meier plots with log-rank test for RFS of HNSCC patients with high vs. low Arg-1 expression levels in tumor parenchyma; divided by recursive partitioning.

The expression levels of Arg-1, i.e., the H score, in the tumor parenchyma and stroma were correlated with clinicopathologic data (Table 1) obtained at the time of surgery (Parenchyma: Figure 2A; Stroma: Supplementary Figure S2A). No significant correlations were observed for either parenchyma or stroma with tumor grade, tumor size or nodal involvement, although there was a trend towards lower parenchyma Arg-1 H scores in patients with nodal metastasis (*p* = 0.064, Figure 2A). However, Arg-1 expression levels in the tumor parenchyma, but not stroma, were significantly different for recurrent and non-recurrent patients (*p* = 0.035, Figure 2A). Recursive partitioning analysis indicated that the H score of 37.5 was a natural cut off for high recurrence risk: 9/13 patients (69%) with H score lower than 37.5 in tumor cells experienced tumor recurrence, whereas only 4/18 patients (22%) with an H score greater than 37.5 recurred (Figure 2B). Thus, the levels of Arg-1 correlated with RFS (Supplementary Table S1) and Kaplan–Meier plots for the RFS of patients with low vs. high Arg-1 expression in tumor cells are shown in Figure 2C. Patients with tumors expressing high Arg-1 protein levels had significantly longer RFS than patients with low levels of Arg-1 expression in tumor cells (*p* = 0.0086). Thus, high Arg-1 levels in tumors emerged as a favorable prognostic factor. Arg-1 expression levels in the stroma did not show any significant association with RFS in patients with HNSCC (Supplementary Figure S2B).

### 3.5. Plasma Levels of Arg-1 Do Not Correlate with Arg-1 Levels in the Tumor

**Figure 3:**
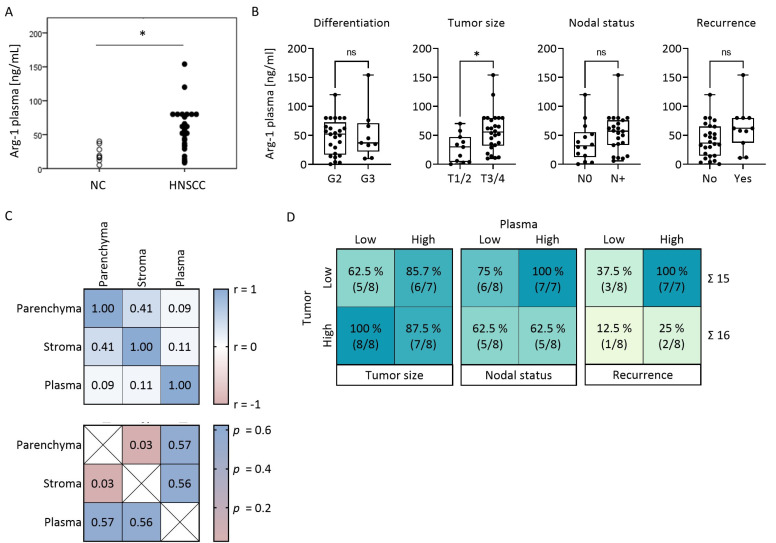
Plasma Arg-1 levels in relation to clinical data and paired tumor tissue. (**A**,**B**) Arg-1 plasma levels measured by ELISA of (**A**) *n* = 10 healthy donors (HD) and *n* = 37 HNSCC patients and (**B**) *n* = 37 HNSCC patients, grouped according to tumor differentiation grade, tumor size, nodal status and development of recurrence. Box-and-whisker blots represent the median, the 25th and 75th quartiles and the range. *p*-values were determined by Mann–Whitney test, with * corresponding to *p* ≤ 0.05 and ns = not significant. (**C**) Spearman correlation matrix between Arg-1 H scores in the tumor parenchyma or stroma and corresponding Arg-1 plasma levels (Spearman r in the upper matrix, *p*-value in the lower matrix). (**D**) 2 × 2 tables comparing corresponding tumor and plasma specimens in 4 groups and in relation to tumor size, nodal status and development of recurrence: Tumor parenchyma Arg-1 low, Tumor parenchyma Arg-1 high, Plasma Arg-1 low, Plasma Arg-1 high; divided by the median. Absolut numbers indicate the number of patients per group.

Plasma samples for Arg-1 ELISA were available from all 37 patients (cohort 1) and 10 healthy donors (HDs). Arg-1 levels were significantly elevated in the plasma of HNSCC patients relative to HDs (*p* < 0.05, Figure 3A). Arg-1 plasma levels did not correlate with the tumor clinicopathological features, such as tumor differentiation, nodal involvement and tumor recurrence (Figure 3B). However, patients with smaller tumors had significantly lower plasma levels of Arg-1 (*p* = 0.011) (Figure 3B). Patients without nodal involvement (*p* = 0.12) or recurrence (*p* = 0.074) tended to have lower plasma levels of Arg-1, but this was not significant (Figure 3B). No correlation was observed between Arg-1 levels in plasma and Arg1 levels in tumor parenchyma or tumor stroma (Figure 3C).

### 3.6. Arg-1 Expression Levels in Paired Tumor and Plasma Specimens

Paired tumor and plasma samples were available from 31 HNSCC patients. Tumor Arg-1 expression levels were high (i.e., above median) in 16 patients (8 with low and 8 with high plasma Arg-1 levels) and were low (i.e., below the median) in 15 patients (8 with low and 7 with high plasma Arg-1 levels) (Figure 3D). Although Arg-1 expression levels in paired tumor and plasma samples did not correlate (Figure 3C), 7/7 (100%) patients with *high plasma* and *low tumor* Arg-1 had nodal metastases and developed recurrence, while only 5/8 (62.5%) and 1/8 (12.5%) patients with *low plasma* and *high tumor* Arg-1 had nodal metastasis and developed recurrence, respectively (Figure 3D). These data emphasize the discrepancy in the clinical significance of tumor-associated vs. soluble plasma Arg-1 and suggest the existence of an unknown factor(s) that independently regulates Arg-1 levels in tumor cells and in plasma.

### 3.7. Plasma-Derived Exosomes in HNSCC Carry Arg-1

**Figure 4:**
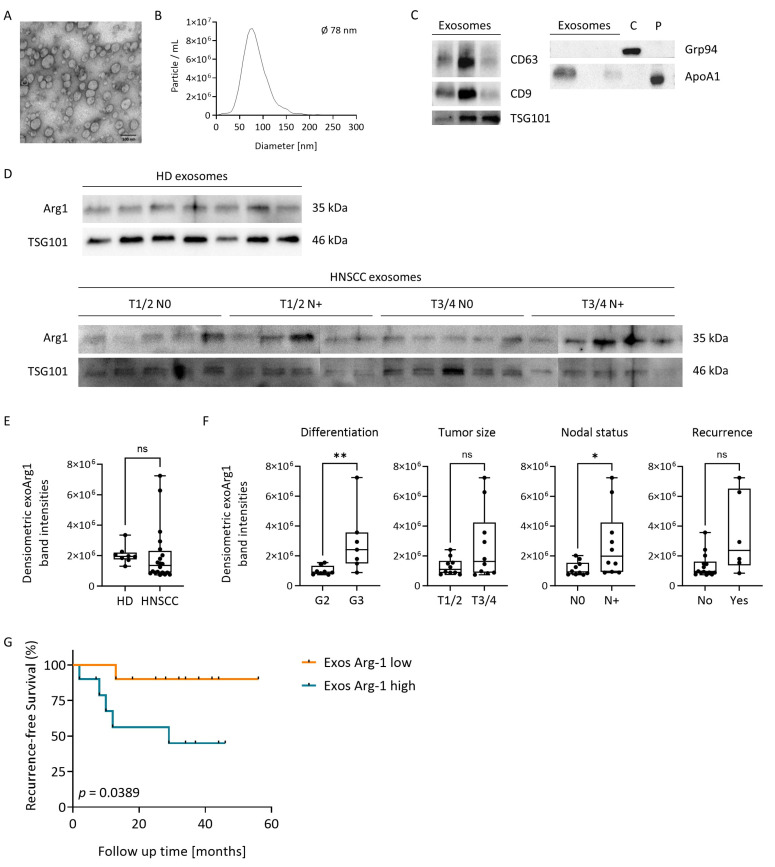
Arg-1 on plasma-derived exosomes in relation to clinical data and RFS. (**A**) Representative transmission electron microscopy image of HNSCC patient’s plasma-derived exosomes. Scalebar = 100 nm. (**B**) Representative size distribution of HNSCC patient’s plasma-derived exosomes measured by nanoparticle tracking. (**C**) Representative Western blot of *n* = 3 HNSCC patients’ plasma-derived exosomes for exosomal markers CD63, CD9, TSG101, the cellular (negative) marker Grp94, and the apolipoprotein Apo1A. Cells (C) and plasma (P) served as positive control for the latter, respectively. (**D**) Arg-1 and TSG101 Western Blots of plasma-derived exosomes from *n* = 8 HD and *n* = 20 HNSCC patients. (**E**,**F**) Arg-1 levels in plasma-derived exosomes, as determined by densiometric analysis (Image Lab software v 6.0.1) from the Western blots in (**D**), from (**E**) *n* = 8 HD and *n* = 20 HNSCC patients and (**F**) *n* = 20 HNSCC patients, grouped according to tumor differentiation grade, tumor size, nodal status and development of recurrence. Box-and-whisker blots show the median, the 25th and 75th quartiles and the range with * *p* ≤ 0.05, ** *p* ≤ 0.001, ns = not significant, as determined by Mann–Whitney test. (**G**) Kaplan–Meier plots with log-rank test for RFS of HNSCC patients with high vs. low Arg-1 levels in plasma-derived exosomes, divided by the median.

Seeking an explanation for the observed discordance between Arg-1 levels in tumor and plasma, we hypothesized that tumor-derived exosomes present in patients’ plasma could be a major but silent (i.e., exosomal Arg-1 cannot be detected when measuring plasma Arg-1 as it is shielded by the exosomal membrane) contributor to high plasma Arg-1 levels in cancer patients. To test this hypothesis, we isolated exosomes from the plasma of 20 HNSCC patients (cohort 2). Transmission electron microscopy confirmed the presence of small vesicles (Figure 4A), ranging in size between 30 and 150 nm (Figure 4B). The vesicles were positive for TSG101, confirming the endosomal origin, and for tetraspanins CD63 and CD9. The cytosolic protein Grp94 was absent, and apolipoprotein, ApoA1, was less abundant in these vesicles compared to pure plasma (Figure 4C). Based on these characteristics, and in agreement with the ISEV criteria, the vesicles are small EVs or exosomes [27]. Arg-1 was present in both exosomes from HDs and HNSCC patients (Figure 4D), with no significant difference between the two groups (Figure 4E). The presence of Arg-1 in plasma-derived exosomes was further confirmed via ELISA upon lysis of exosomes, although the detection levels were very low (Supplementary Figure S3). Importantly, when grouping HNSCC patients according to their clinicopathology, exosomal Arg-1 levels were significantly elevated in patients with poorly differentiated tumors (*p* = 0.009) and nodal metastasis (*p* = 0.023) (Figure 4F). Also, exosomal Arg-1 levels tended to be elevated in patients developing recurrence (*p* = 0.051) (Figure 4F). Despite the small patient cohort, Kaplan–Meier analysis for RFS revealed a significant association of high exosomal Arg-1 levels with worse RFS, while patients with low Arg-1 levels in exosomes had longer RFS (*p* = 0.0389, Figure 4G). Overall, high Arg-1 levels in exosomes obtained from the plasma of HNSCC patients emerged as indicators of nodal metastasis and unfavorable prognosis.

## 4. Discussion

Tumor cells are known to produce a variety of immunosuppressive cytokines and other soluble factors [29]. Late-stage HNSCCs with nodal metastasis create a strongly suppressive microenvironment, in which the functions of immune cells are blocked or eliminated [5]. Tumor escape from the host immune system has often been linked to the presence of immunosuppressive factors, such as Arg-1, in the TME. Arg-1 is recognized as both a promoter of tumor growth and inhibitor of anti-tumor immunity [8,12]. It catalyzes the hydrolysis of arginine to ornithine and urea, thereby depleting arginine, an essential amino acid for T-cell proliferation, from the TME, thus inhibiting T-cell responses and promoting tumor immune escape [30]. Arg-1 is produced by many solid tumors and by various subsets of immune as well as tissue cells [31,32,33]. Most studies to date focused on Arg-1 released by myeloid cells, MDSCs or tumor-associated macrophages, while its role in cancer cells remains largely unclear [34,35].

We investigated Arg-1 levels in tumor tissue and the circulation (plasma, exosomes) of HNSCC patients in relation to clinical data. Although the involvement of Arg-1 in tumorigenesis is indisputable, its association with clinicopathological data and its prognostic impact remain largely inconsistent among different tumors and biological fluids analyzed. For example, elevated *serum/plasma* Arg-1 activity was reported to correlate to the extent of metastasis and poor prognosis in breast cancer patients [36,37]. Others reported that Arg-1 expression in breast cancer *tissue* was positively correlated to better patients’ prognosis, suggesting that Arg-1 plays a tumor-suppressor role [38]. In contrast, tumor Arg-1 expression in colorectal cancer was associated with high-stage tumors, lymph node metastases and reduced survival [39]. In HNSCC patients, Arg-1 mRNA expression in the tumor was reported to be a negative prognostic factor for overall survival [40]. This, however, could not be confirmed with the TCGA cohort, where gene expression levels of Arg-1 in the tumor had no impact on the overall survival of HNSCC patients [28]. Elevated soluble Arg-1 levels were previously detected in HNSCC, breast, lung or colon cancer patients [36,37,41,42,43].

Here, we report that in HNSCC patients, high *tumor* Arg-1 protein expression was associated with favorable clinicopathological data and longer RFS, while high plasma levels of soluble Arg-1 were associated with unfavorable clinicopathological data. These divergent results suggest a context-dependent regulation of Arg-1, i.e., tumor-associated Arg-1 and soluble Arg-1 in plasma may be differently and independently regulated. When examining paired samples of HNSCC tissue and plasma, an additional discrepancy was discovered: all patients with *low tumor* and *high plasma* Arg-1 levels had nodal metastases and developed recurrence. This result suggested that aggressive tumors may contain low Arg-1 levels because they export Arg-1 into the periphery, where it facilitates T-cell impairment and systemic immune suppression. The production and release of tumor-derived exosomes (TEX) represent a way for the Arg-1 export from the tumor cells to peripheral tissues or blood. Thus, TEX represent a highly efficient mechanism of tumor-orchestrated immune evasion.

Arg-1^+^ exosomes were previously described in the context of ovarian cancer [44] and glioblastoma [45] but not of HNSCC. Ascites-derived and plasma-derived exosomes containing Arg-1 were found to suppress T-cell responses and promote tumor progression in ovarian carcinoma [44]. We previously reported that Arg-1^+^ exosomes produced by in vitro reprogrammed tumor-associated macrophages (TAMs) promoted glioblastoma growth [45]. To the best of our knowledge, we are the first to report that plasma-derived exosomes from HNSCC patients carry Arg-1, thereby supporting our hypothesis of Arg-1 export from the tumor to plasma via exosomes. As HD exosomes carried similar levels of Arg-1 compared to HNSCC exosomes, tumor cells cannot be the only cells releasing Arg-1 via exosomes. Indeed, Arg-1 is involved in a variety of physiological biochemical processes [46], and we cannot exclude the contribution of other cell types to exosomal Arg-1 levels through the analysis of total plasma exosomes. However, there is no study comparing the levels of exosomal Arg-1 in physiological and oncological settings so far. It was described that diet and fasting plasma glucose levels can induce changes in exosomal Arg-1 levels [47,48], and these factors were not possible to consider in selecting our HD cohort. Further, the contribution of different cell types to Arg-1 levels in plasma exosomes could change with the onset of tumorigenesis, with a higher relative amount of tumor cells releasing Arg-1 exosomes while keeping total exosomal Arg-1 levels at the same level as in non-tumor individuals. Despite there being no difference between exosomal Arg-1 levels of HDs and HNSCC patients, the levels within the HNSCC patient group showed significant differences depending on the presence of lymph node metastasis, providing evidence for the possible usage of exosomes as markers for HNSCC tumor aggressiveness.

In the context of HNSCC, tumor cells appear to package Arg-1 into exosomes and transport it to the periphery and to distant cells, thereby regulating Arg-1 levels in the producing tumor cells. The consequences of this communication mechanism are two-fold: tumors can remove Arg-1 from the primary microenvironment using exosomes to (a) induce systemic immune suppression and (b) prepare distant lymph nodes for metastasis, thereby promoting tumor growth and the development of recurrence. This way, exosomes from patients with high-stage tumors and lymph node metastases would be assumed to contain higher levels of Arg-1 compared to patients without nodal involvement. Indeed, Arg-1 was present at higher levels in exosomes from the plasma of HNSCC patients with positive lymph nodes compared to N0 patients. However, the levels of soluble Arg-1 in the plasma of these HNSCC patients do not reflect the presence of Arg-1 sequestered in exosomes and underestimate the true levels of circulating Arg-1. As the half-life of free Arg-1 in humans is <30 min [49,50], it can only reflect a current and fragile snapshot of circulating Arg-1. In contrast, intra-exosomal Arg-1 is expected to be protected from degradation by the exosomal membrane, providing a stable transport of the functionally active enzyme. Indeed, recombinant Arg-1 tested in vivo failed to inhibit antigen-specific T-cell proliferation, whereas Arg-1^+^ exosomes were able to do so [44]. Similarly, IHC of the tumor likely underestimates true Arg-1 levels, because it does not account for the active exosome release, which is presumably enhanced in metastasizing, aggressive tumors.

## 5. Conclusions

In conclusion, neither IHC in situ nor measurements of soluble Arg-1 in plasma adequately reflects the biologically active Arg-1 levels in metastatic HNSCC. Exosomes emerge as potentially highly accurate biomarkers of lymph node metastases in HNSCC patients. Arg-1 carried by exosomes provides a new mechanism for the intercellular transport of an enzyme which is critical for metastasis formation. Further studies are needed to confirm the potential role of Arg-1 in exosomes as a biomarker for lymph node metastases and RFS in HNSCC and other solid tumors.

## Figures and Tables

**Table 1:** Demographic and clinicopathologic characteristics of the HNSCC patients included in this study ^1^.

Characteristics	Cohort 1 (Tumor, Plasma) No. (%) of *n* = 37	Cohort 2 (Exosomes) No. (%) of *n* = 20
Age in years		
Median	60	65
Range	47–78	51–82
Gender		
Male	29 (78)	12 (60)
Female	8 (22)	8 (40)
Histological differentiation		
G1	1 (3)	2 (10)
G2	25 (68)	8 (40)
G3	9 (24)	7 (35)
N/A	2 (5)	3 (15)
Tumor size (T)		
T1	7 (19)	4 (20)
T2	4 (11)	6 (30)
T3	13 (35)	6 (30)
T4	13 (35)	4 (20)
Nodal status (N)		
N0	14 (38)	10 (50)
N1	8 (22)	4 (20)
N2	12 (32)	2 (10)
N3	3 (8)	4 (20)
Distant metastasis (M)		
M0	37 (100)	20 (100)
UICC 8th edition		
I/II	11 (30)	12 (60)
III/IV	26 (70)	8 (40)
Recurrence		
No	24 (65)	14 (70)
Yes	13 (35)	6 (30)

^1^ Patients were evaluated at the time of surgery.

## Data Availability

All data relevant to the study are included in the article or uploaded as Supplementary Information.

## Supplementary Materials

The following supporting information can be downloaded at: https://www.mdpi.com/article/10.3390/cancers15225449/s1, Table S1: Arg-1 H scores for patients’ tumors and recurrence status. Figure S1: Spearman correlation analysis of Arg-1 H scores between tumor parenchyma and tumor stroma. Figure S2: Stroma Arg-1 expression in relation to clinical data and recurrence free survival (RFS). Figure S3: Arg-1 levels in plasma-derived exosomes, as determined by ELISA assay upon exosome lysis. Figure S4: Arginase-1 and TSG101 original Western blots. Table S2: Background-corrected densitometry readings of Arginase-1 Western blot.
