# Supplementary material for: Arginase-1 in Plasma-Derived Exosomes as Marker of Metastasis in Patients with Head and Neck Squamous Cell Carcinoma
Source: Cancers (Basel). 2023 Nov 16;15(22):5449. doi: 10.3390/cancers15225449 (PMC10670520; doi:10.3390/cancers15225449)
Supplement: Supplementary file 1 [file cancers-15-05449-s001.zip › cancers-2620506-supplementary.pdf]

cancers

MDPI

Supplementary information for manuscript

"Arginase-1 in plasma-derived exosomes as marker of metastasis in patients with head and neck squamous cell carcinoma"

Supplementary Table S1. Arg-1 H scores for patients' tumors and recurrence status.[1]

Patient # (Cohort 1)	Tumor Parenchyma	Tumor Stroma	Tumor Recurrence
1	150	75	No
2	25	ND	Yes
3	0	0	Yes
4	300	0	No
5	225	0	No
6	25	0	Yes
7	300	225	Yes
8	75	225	No
9	25	0	Yes
10	25	0	No
11	25	0	Yes
12	75	0	Yes
13	75	25	No
14	75	100	No
15	50	0	Yes
16	150	25	No
17	150	0	No
18	25	0	Yes
19	150	150	No
20	25	225	Yes
21	75	150	No
22	75	225	Yes
23	0	0	No
24	150	100	No
25	25	0	Yes
26	150	0	No
27	0	0	No
28	50	0	No
29	200	100	No
30	25	25	Yes
31	0	0	No

[1] The H scores for the tumor parenchyma and the tumor stroma were calculated as described in Materials and Methods and are based on the IHC results in tissue sections of tumor specimens removed at surgery. Tumor recurrence within 4 years of last therapy is indicated.

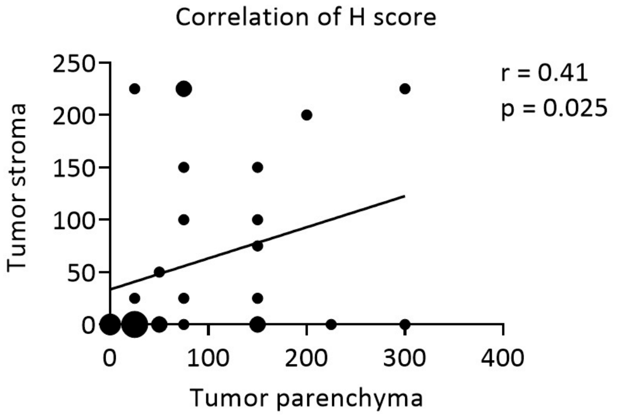

Supplementary Figure S1. Spearman correlation analysis of Arg-1 H scores between tumor parenchyma and tumor stroma. r = Spearman correlation coefficient.

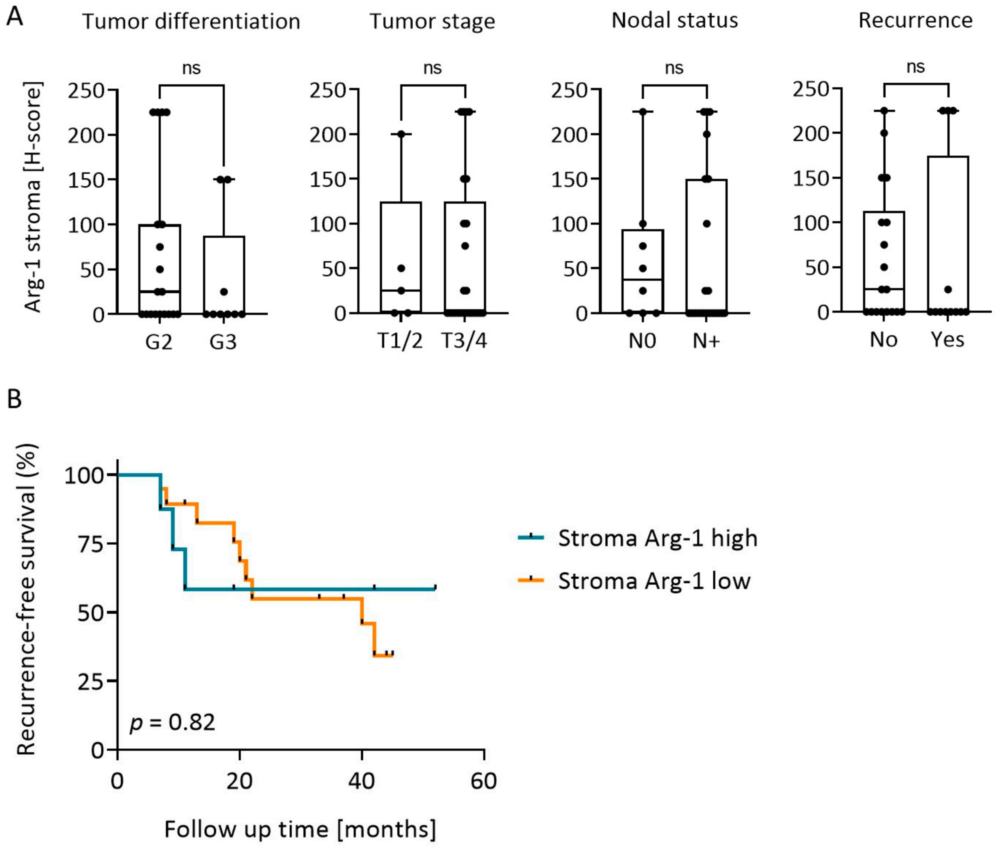

Supplementary Figure S2. Stroma Arg-1 expression in relation to clinical data and recurrence free survival (RFS). **(A)** Arg-1 H scores in tumor stroma of n = 31 HNSCC patients, grouped according to tumor differentiation grade, tumor size, nodal status and development of recurrence. P-values were determined by Mann–Whitney test, with ns = not significant. **(B)** Kaplan-Meier plots with log-rank test for RFS of HNSCC patients with high vs low Arg-1 expression levels in tumor parenchyma.

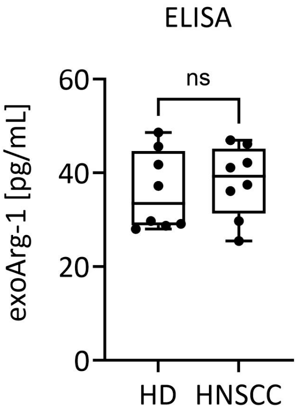

Supplementary Figure S3. Arg-1 levels in plasma-derived exosomes, as determined by ELISA assay upon exosome lysis, from n = 8 HD and n = 8 HNSCC patients. P-values were determined by Mann–Whitney test, with ns = not significant.

Arginase-1 original Western Blots, exposure time 14.1 sec

1.

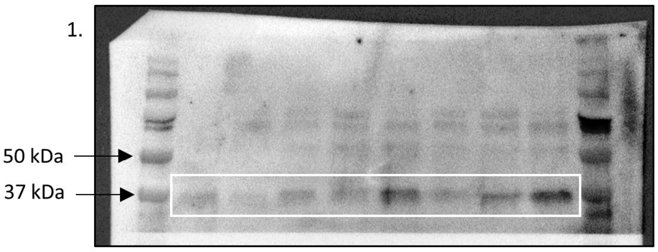

Lane 1 & 10:
Precision Plus Protein Dual Color Standards, Bio-Rad

Lanes 2 to 9:
Exosome samples #1 to 8

2.

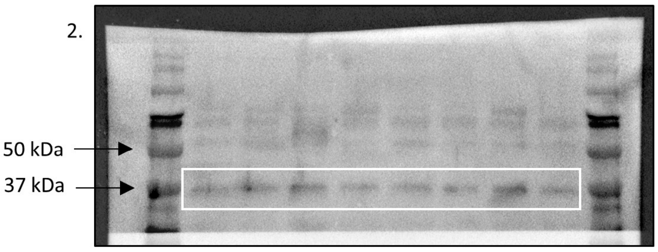

Lane 1 & 10:
Precision Plus Protein Dual Color Standards, Bio-Rad

Lanes 2 to 9:
Exosome samples #9 to 16

3.

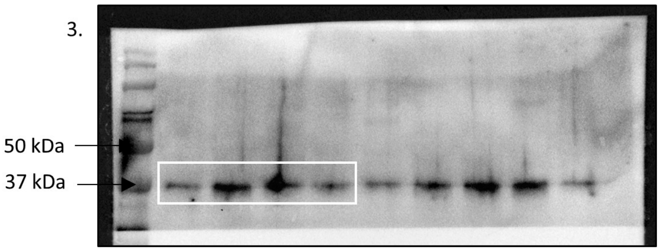

Lane 1:
Precision Plus Protein Dual Color Standards, Bio-Rad

Lanes 2 to 5:
Exosome samples #17 to 20

Lanes 6 to 10:
Samples not relevant to this study.

TSG101 original Western Blots, exposure time 27 sec

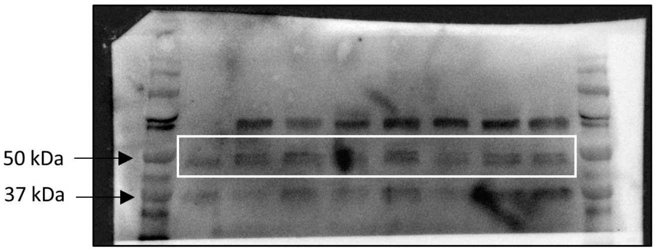

Lane 1 & 10:
Precision Plus Protein Dual Color Standards, Bio-Rad

Lanes 2 to 9:
Exosome samples #1 to 8

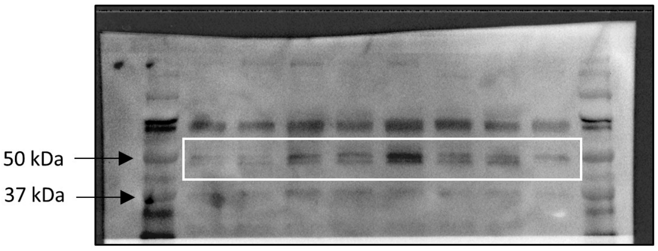

Lane 1 & 10:
Precision Plus Protein Dual Color Standards, Bio-Rad

Lanes 2 to 9:
Exosome samples #9 to 16

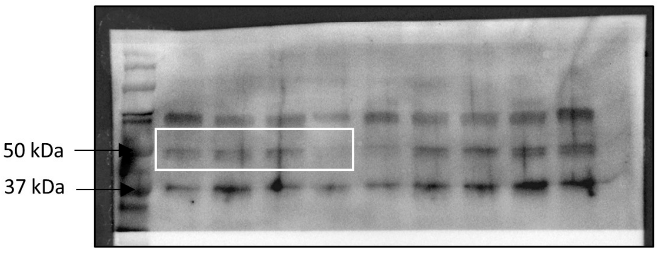

Lane 1:
Precision Plus Protein Dual Color Standards, Bio-Rad

Lanes 2 to 5:
Exosome samples #17 to 20

Lanes 6 to 10:
Samples not relevant to this study.

Supplementary Figure 4. Arginase-1 and TSG101 original Western Blots.

Supplementary Table 2. Background-corrected densitometry readings of Arginase-1 Western Blot.

Patient # (Cohort 2)	Arginase-1 band intensity
HNSCC	
1	884583
2	755055
3	764631
4	1249605
5	2026017
6	890190
7	1488501
8	2414538
9	939834
10	1548162
11	1458261
12	841680
13	968940
14	738675
15	1796382
16	880110
17	2937690
18	6277257
19	7246260
20	3568194
HD	
1	1931462
2	1719900
3	2047500
4	1967212
5	1842906
6	2254434
7	1305824
8	3348852
